# Insulin-Like Growth Factor-1 in Acute Ischemic Stroke

**DOI:** 10.1186/s41983-018-0042-y

**Published:** 2018-12-10

**Authors:** Hala Shaheen, Sayed Sobhy, Sherine El Mously, Manal Niazi, Mohammed Gomaa

**Affiliations:** Faculty of Medicine, Fayoum University, Keman Fares area, Fayoum City, 63611 Egypt

**Keywords:** Stroke, Risk factors, IGF-1

## Abstract

**Background:**

Cerebrovascular ischemic stroke is highly prevalent in the general population and is considered one of the frequent causes of mortality and disability. Insulin-like growth factor-1 (IGF-1) is recognized as an important neuro-protective factor against cerebral vascular ischemic insult.

**Aim of the work:**

To study the relationship between serum IGF-1 levels and acute ischemic stroke (AIS) in the Egyptian population.

**Patients and methods:**

Two hundred patients with AIS (within the first 24 h) were subjected to full neurological examination, assessment of stroke severity using National Institute of Health Stroke Scale (NIHSS) and measurement of serum IGF-1 levels. The control group included 100 subjects matched for age, gender, and conventional vascular risk factors.

**Results:**

Serum IGF-1 levels were significantly reduced in cases of first AIS compared to control group. A reduced serum IGF-1 level was an independent risk factor for ischemic stroke with cut off value less than 148.3 ng/ml associated with increased AIS risk.

**Conclusion:**

Lower IGF-1 levels are significantly related to risk of ischemic stroke occurrence, independent from other conventional risk factors in the Egyptian population.

## Introduction

Cerebrovascular stroke is one of the most frequent causes of death and disability worldwide; it has significant clinical and socioeconomic impact [1].

The expanding use of biomarkers in the field of stroke has made a substantial impact in our understanding of the pathophysiology of stroke and the treatment approach [2].

Insulin-like growth factor-1 (IGF-1) is a single-chain polypeptide hormone composed of 70 amino acids; it shares homology with proinsulin. It is produced mainly by the liver in response to the endocrine growth hormone (GH) stimulus, but it is also secreted by multiple tissues for autocrine / paracrine purposes [3].

IGF-1 has a pivotal role in the development, cell differentiation, plasticity and survival of the nervous system [4]. IGF-1 exerts neuroprotective effects in both white and gray matter under different detrimental conditions. It is a key regulator of cell proliferation and an inhibitor of cell apoptosis and necrosis [5].

In the last decade, numerous studies have investigated the effect of IGF-1 concentration on aging and different aging-related diseases [6].

Several previous studies showed that low-normal levels of IGF associated with increased mortality in ischemic heart disease and stroke [7]. Thus, we thought to investigate the significance of serum IGF-1 levels in a cohort of Egyptian patients with acute ischemic stroke (AIS). (GH is the major regulator of total IGF-116; aging is associated with a decline in GH. These facts explain why IGF normally decline significantly with age. It is recommended that each lab should determine its own normal and abnormal values according to kits used in this study (DRG IGF-1600 ELISA Kit (Reference # EIA-4140) mean level was (176.3 ng\ml in age group 40–59 years and 137.2 ng\ml in age group 60–80 years.

## Subjects and methods

This is a case control cross-sectional study conducted on 200 Egyptian patients presented AIS, within the first 24 h. Patients were recruited from the Neurology Departments in Fayoum University Hospital, Fayoum General Hospital and Fayoum Health Insurance Hospital in the period from April 2015 to February 2017. Patients were defined according to the World Health Organization (WHO) criteria [8] and had symptom onset within 24 h. We excluded patients with Parkinson disease, recurrent ischemic stroke, intra-cerebral hemorrhage, medical illness, or current medications that influence serum IGF-I levels such as dwarfism, acromegaly, chronic kidney diseases, liver diseases, and high dose of estrogen. We excluded also patients suffering from congestive heart failure, malignant tumor, renal insufficiency, severe edema, febrile disorders, systemic infections, history of recent surgery, or trauma during the preceding 2 months and autoimmune diseases.

One hundred adults were enrolled in our study as a control group. They came to the neurology clinic seeking medical advice for headache or spondylosis. They were matching the cohort group in age, sex, and conventional vascular risk factors.

All patients and controls were subjected to detailed history taking, general and full neurological examination, assessment of severity of neurological deficit of stroke according to National Institute of Health Stroke Scale (NIHSS) scores ranging from 0 to 42; with higher values reflecting more severe neurologic damage [9]. Stroke subtypes were classified according to Trial of Org 10,172 in Acute Stroke treatment (TOAST) criteria [10] that denote 5 subtypes of ischemic stroke: large-artery atherosclerosis, cardio-embolism, small-vessel occlusion, stroke of other determined etiology, and stroke of undetermined etiology. To confirm the diagnosis of AIS, all patients underwent a magnetic resonance imaging (MRI) with diffusion scan (stroke protocol) on Titan Toshiba 1.5 Tesla of the brain in the Radiology Department, Fayoum University Hospital.

Routine laboratory investigations were also performed including complete blood count (CBC), serum C-reactive protein (CRP) level, erythrocyte sedimentation rate (ESR), liver and kidney function tests to exclude the presence of systemic or metabolic disorders, fasting blood glucose level, and finally serum cholesterol.

Regarding the serum IGF-1, venous blood samples were collected within the first 24 h of the AIS in the fasting state. We collected 5 ml of venous blood in vacutainer collection tubes. Samples were left to clot for 30 min before centrifugation; the latter was applied for 15 min at approximately 1500×*g*. Serum was removed and assayed immediately or stored at − 80 °C until analysis.

IGF-1 was measured using DRG IGF-1600 ELISA Kit (Reference # EIA-4140). The kit is a solid phase enzyme-linked immunosorbent assay (ELISA) based on the principle of competitive binding. Patient samples, standards, and controls were acidified and neutralized prior to the assay procedure. The micro titer wells were coated with a monoclonal antibody directed towards an antigenic site on the IGF-1 molecule. The pre-treated sample was incubated at room temperature with the conjugate (biotinylated IGF-1). The wells were washed then incubated with enzyme complex (Streptavidin-HRP-complex). After addition of the substrate solution, the concentration of IGF-1 was calculated in a reverse proportional way to the intensity of the color developed.

### Ethical consideration

The study was approved by the Faculty of Medicine, Fayoum University Research Ethical Committee. All participants either patients or controls were informed about the objectives of the study, the examination, and the investigations. The confidentiality of their information and their right not to participate in the study were considered. Written informed consents were obtained from all of them.

Date of ethical committee15/4/2015.

Session number-13 N (D58).

### Statistical analysis

Data were collected and coded into Microsoft Access, and data analysis was performed using SPSS software version 18 in windows 7. Simple descriptive analysis was performed in the form of numbers and percentages for qualitative data. Quantitative data were described in the form of arithmetic means, standard deviation (SD), and range. Independent Student’s *t* test was used to compare two independent groups of quantitative data as age and serum level of IGF-1. Chi-square test was used to compare qualitative groups such as sex. Bivariate (Pearson) correlation test was used to assess the association between variables. The level *p* < 0.05 was considered the cut-off value for significance.

## Results

The mean age of the patients group was 58.7 ± 3.6 years ranging between 53 and 71 years. The control subjects’ mean age group was 58.4 ± 3.9 years ranging between 50 and 68 years. The sex distribution among the patients was 45% females and 55% males versus 44% females, and 56% males among control groups. Hypertension and hypercholesterolemia were the most encountered risk factors among patients and control groups (Table 1). There were no statistically significant differences (*p* > 0.05) between cases and control as regard age, (sex) and conventional risk factors.

**Table 1:** vascular risk factors among stroke and control group

Risk factors	Cases number	Controls
Overweight	87 (43.5%)	41 (41%)
Hypertension (*N*, %)	107 (53.5%)	52 (52%)
High cholesterol (*N*, %)	82 (41%)	39 (39%)
Ischemic heart disease (*N*, %)	62 (31%)	31 (31%)
Diabetes mellitus (*N*, %)	60 (30%)	30 (30%)
Atrial fibrillation (*N*, %)	46 (23%)	20 (20%)
Smoking (*N*, %)	44 (22%)	19 (19%)

*N* number, % percent

One hundred seventy four patients (87%) presented with manifestations of anterior circulation stroke such as hemiparesis and hemihypothesia, while 26 patients (13%) presented with manifestations of posterior circulation stroke such as crossed hemiplegia, ataxia, and vertigo. Regarding the etiology of stroke, 82 (41%) patients had small vessel disease, 58 (29%) had large vessel disease, 49 (24.5) had cardio-embolic stroke, 6 (3%) had ischemic stroke of other determined etiology, and 5 (2.5%) had stroke of undetermined etiology. The mean NIHSS of stroke cases was (8.9 ± 0.58) ranged between 1 and 30.

The mean serum IGF-1 among stroke cases was (143.4 ± 12.6) ng/ml ranged between 98.6 and 173.9 ng/ml while among control group it was (156 ± 11.2) ng/ml ranged between 137.5 and 179.2 ng/ml. There was a statistically significant difference (*p* < 0.001) between the study groups regarding the serum IGF-1 with low mean among stroke cases. Sensitivity and specificity test for IGF-1 in diagnosis of stroke illustrated that the probability of being true positive was (78.8%) more than being false positive when repeat test 100 times with sensitivity (70%) and specificity (72%) with cut-off 148.3 ng/ml.

There was no statistically significant difference (*p* > 0.05) in IGF-1 serum levels between males and females among the study groups (Table 2).

**Table 2:** comparison of IGF-1 between males and females among the study groups

Sex	IGF 1		*p*
	Mean	SD	
All study group			
Females	148.5	12.2	0.3
Males	146.9	14.4	
Among cases			
Females	144.3	11.3	0.4
Males	142.7	13.5	

*IGF-1* insulin growth factor-1

**Fig. 1:**
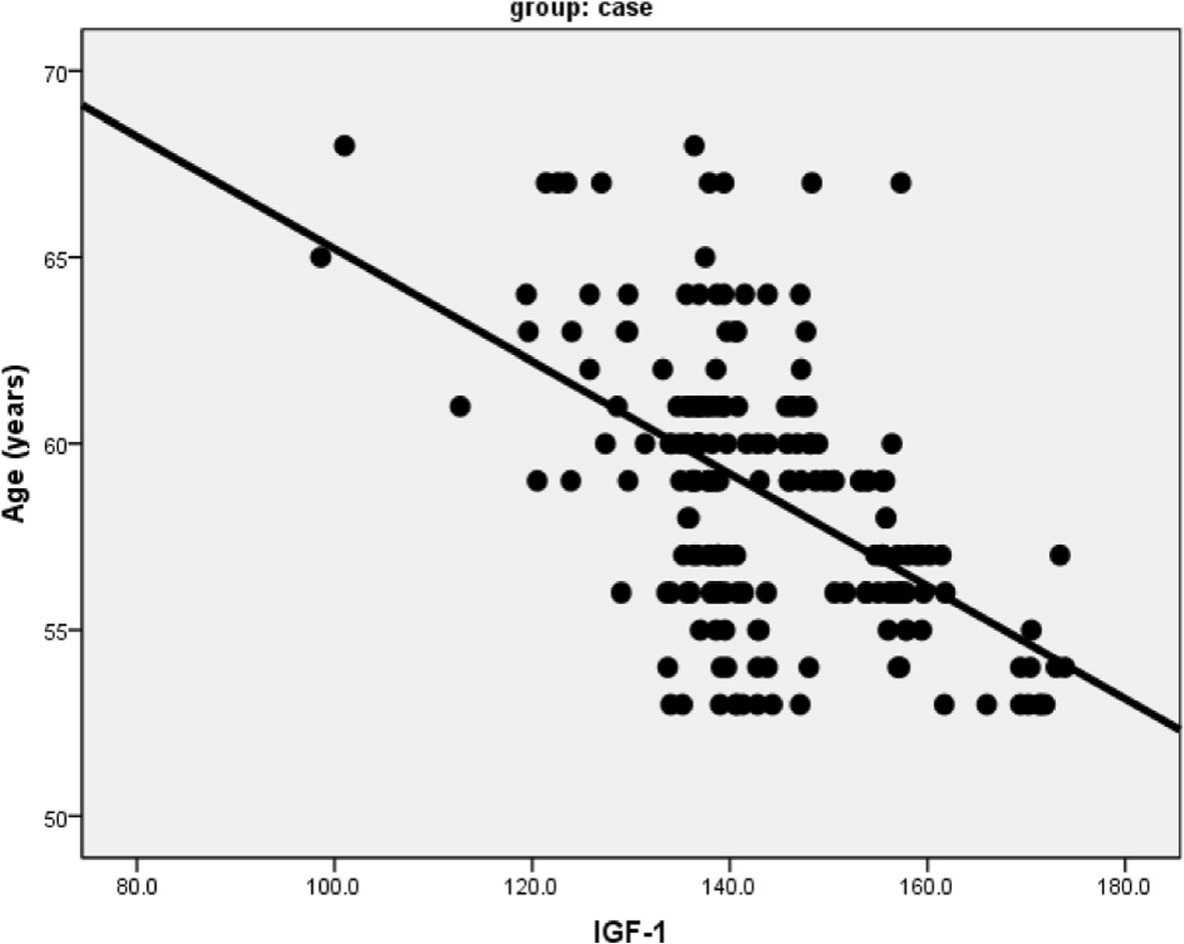
Correlation between IGF-1 and age of patients. IGF-1, insulin growth factor-1

There was a statistically significant negative correlation (*r* − 0.52; *p* < 0.001) between IGF-1 serum levels of patients and age (Fig. 1); while no statistically significant correlation was detected (*r* − 0.03; *p* > 0.6) between IGF-1 and stroke severity measured by NIHSS. (stroke cases divided to anterior and posterior circulation and no significant differences found as regard severity measured by NIHSS >> > …serum level measured and studied according to TOAST classification and no statistically significant differences were found.

## Discussion

Our study revealed that serum IGF-1 levels were significantly reduced in cases of first AIS compared to controls. For the entire group, after adjusting other possible risk factors, a reduced IGF-I level was an independent risk factor for stroke with cut off value less than 148.3 ng/ml associated with increase AIS risk. This result was in concordance with Dong X and colleagues [11] who found that the lower IGF-1 levels of less than 135 ng/ml were significantly related to risk of stroke, independent from other traditional and emerging risk factors. They suggested that IGF-1 may play a role in the pathogenesis of AIS; (as insufficient IGF-1 levels play a role in vascular diseases such as atherosclerosis and restenosis, atherosclerotic plaque involves many factors and IGF-1 have a vital role in its formation evident by presence of its receptors on smooth muscle cells, inflammatory cells, and arterial endothelial cells of atherosclerotic plaques) so, should be considered as a routine risk factor for stroke in the Chinese population. This difference in cut-off value can be attributed to racial differences between Egyptian and Chinese population.

Another study published by Hamidreza and colleagues [12] showed that circulating IGF-1 levels were associated with risk of ischemic stroke, with subjects in the lowest quintile of IGF-1 levels having a 2.5-fold higher risk of incident ischemic stroke. This association remained significant after adjusting the traditional stroke risk factors. Their results suggest that IGF-1 is related to risk of incident ischemic stroke and support an emerging hypothesis that low circulating IGF-1 may be an important determinant of ischemic stroke events. On the other hand, Robert and colleagues [13] found that total IGF-1 levels did not predict risk of incident ischemic stroke. This result can be explained by the exerted effects of IGF-1 on nitrogen oxide production, plaque stability, anti-inflammatory actions, increased endothelial cell survival, and inhibition of endothelial cell apoptosis; all those effects directly opposed the development of ischemic stroke [14].

Our results showed a statistically significant negative correlation between IGF-1 and age indicating that an increase in age is associated with decrease in IGF-1 levels. This result agrees with the data shown by Dong X and colleagues [11] and Roubenoff R and colleagues [15].

GH is the major regulator of total IGF-1 [16]; with advanced age, there is a decrease in muscle and an increase in adiposity associated with a decline in GH [17]. These facts explain why IGF significantly decline with age in normal population.

There was no statistical difference in IGF-1 levels between males and females which is concordant with previous studies [18, 19]. Nevertheless, there was no statistically significant correlation between IGF-1 levels and stroke severity assessed by NIHSS which agrees the results of De Smedt and colleagues [20] who found that the baseline stroke severity did not differ between high- and low-IGF-1 groups. However, other studies [11, 21] showed an inverse correlation between IGF-1 and stroke severity. IGF-1 suppresses apoptosis through mechanisms involving activation of multiple protein kinase pathways, which are important in the latent phase of evolving programmed cell death [22]. This may explain why we found no statistically significant correlative differences in baseline stroke severity between patients with high and low IGF-1 serum levels (correlation between stroke severity and serum level).

Some limitations should be considered in our study. First, serum IGF-1 levels were only measured once after stroke onset, and additional measurements in the days after would have been of interest. Second, biologically active free IGF-1 and IGF-binding proteins were not measured. It is suggested that levels of IGF-binding proteins may affect the actions of circulating IGF-1 [23]. Yet, circulating IGF-1 is highly correlated with the ratio of IGF-1 to IGF-binding proteins [24]. Therefore, measuring circulating IGF-1 alone may lead to underestimation of true relationships with incident stroke. Finally, this study included older Egyptian patients that may limit generalization to other ethnicities or younger populations.

## Conclusions and recommendations

Lower serum IGF-1 levels are significantly related to risk of stroke, independent from other traditional and emerging risk factors. AIS is more likely to occur in patients with low serum IGF-1 levels in the Egyptian population. We recommend a routine screening of serum IGF-1 levels in patients having different stroke risk factors as a preventive measure. However, before a broad implementation, additional studies are needed for external validation. Further studies are warranted to explore the underlying cellular mechanisms and investigate potential clinical implications of IGF-1. Moreover, further studies are required to clarify the neuroprotective mechanisms of IGF-1 in ischemic stroke process. Enhancing serum IGF-1 levels may be an interesting target to be considered in future therapeutic strategies via performing placebo-controlled trials to increase serum IGF-1 levels in patients with AIS.

## Abbreviations

AIS: Acute ischemic stroke
BMI: Body mass index
CBC: Complete blood count
CRP: C-reactive protein
DRG IGF-1600 ELISA: Dorsal root ganglion IGF-1600 ELISA
ELISA: Enzyme-linked immunosorbent assay
ESR: Erythrocyte sedimentation rate
GH: Growth hormone
IGF-1: Insulin-like growth factor-1
MRI: Magnetic resonance imaging
NIHSS: National Institute of Health Stroke Scale
SD: Standard deviation
Streptavidin-HRP-Complex: Streptavidin-horse radish protein complex
TOAST: Trial of Org 10,172 in acute stroke treatment
WHO: World Health Organization
